# Diet and Hygiene in Modulating Autoimmunity During the Pandemic Era

**DOI:** 10.3389/fimmu.2021.749774

**Published:** 2022-01-05

**Authors:** Leila Abdelhamid, Xin M. Luo

**Affiliations:** ^1^ Department of Biomedical Sciences and Pathobiology, College of Veterinary Medicine, Virginia Tech, Blacksburg, VA, United States; ^2^ Department of Microbiology, College of Veterinary Medicine, Alexandria University, Alexandria, Egypt

**Keywords:** diet, hygiene, immunomodulation, immune homeostasis, autoimmunity, COVID-19

## Abstract

The immune system is an efficiently toned machinery that discriminates between friends and foes for achieving both host defense and homeostasis. Deviation of immune recognition from foreign to self and/or long-lasting inflammatory responses results in the breakdown of tolerance. Meanwhile, educating the immune system and developing immunological memory are crucial for mounting defensive immune responses while protecting against autoimmunity. Still to elucidate is how diverse environmental factors could shape autoimmunity. The emergence of a world pandemic such as SARS-CoV-2 (COVID-19) not only threatens the more vulnerable individuals including those with autoimmune conditions but also promotes an unprecedented shift in people’s dietary approaches while urging for extraordinary hygiene measures that likely contribute to the development or exacerbation of autoimmunity. Thus, there is an urgent need to understand how environmental factors modulate systemic autoimmunity to better mitigate the incidence and or severity of COVID-19 among the more vulnerable populations. Here, we discuss the effects of diet (macronutrients and micronutrients) and hygiene (the use of disinfectants) on autoimmunity with a focus on systemic lupus erythematosus.

## Introduction

The immune system is highly toned and efficiently dedicated to maintaining health by protecting against a tremendous array of invaders (1). Our body faces continuous challenges that might be represented in biological threats (such as, but not limited to, microbial pathogens) and physical threats (such as radiation and chemicals). The immune system is equipped with both innate and adaptive arms to fight against these threats (1). The ultimate goal of our immune system is to induce an effective and balanced inflammatory response that enables the efficient elimination of possible threats without causing excessive collateral tissue damage (2). Therefore, an optimal immune response includes recognition, mounting an overarching reaction, then returning to normal homeostasis without skewing to either immunodeficiency or autoimmunity (3, 4).

It is fascinating how this system discriminates between friends and foes to maintain homeostasis (5).

Innate defenses are inborn abilities of the immune system to detect, attack, and eliminate (or at least restrain) pathogenic invaders. Part of the body’s defense system that includes physical barriers (6), antimicrobial peptides (7), and complement proteins (8), innate immune cells are equipped with germline-encoded sensors called pattern recognition receptors (PRRs), which are predestined to recognize highly conserved molecular patterns including both pathogen- and damage-associated molecular patterns (PAMPS and DAMPs, respectively) (9, 10). Recognition of these molecular patterns allows for rapid host defense while “mostly” maintaining nonreactivity to self (thorough recognizing only PAMPs and DAMPs) (9, 10). This also paves the way for adaptive immune recognition that provides long-lasting immunity (11). Indeed, cellular components of the innate system, namely antigen-presenting cells (APCs) such as macrophages and dendritic cells (DCs) (12–14), as well as neutrophils (15), bridge the interface between innate and adaptive responses. APCs, in particular, play pivotal roles in informing their adaptive counterpart (T and B lymphocytes) (16) for mounting antigen-specific responses with long-term memories that could also protect against future threats. The adaptive immune cells are highly antigen-specific (17, 18) and in a way to maintain self-tolerance, these cells cannot be activated solely by the initial recognition of antigen peptides through their somatically rearranged receptors (T cell receptor and B cell receptor, or TCRs and BCRs, respectively); instead, their activation also requires costimulatory and cytokine signals in addition to antigen recognition (19–21). The co-stimulation signals ensure nonreactivity when encountering self-antigens, in which case peripheral tolerance (through deletion or anergy) would be induced (22–24). However, it is complicated how deviations would occur and specifically, how the breach of self-tolerance and autoimmunity would develop.

According to the National Stem Cell Foundation, nearly 4% of the world’s population are affected by one or more autoimmune disorders; and as of 2019, the National Institute of Health estimated that around 7% of adults in the United States had been diagnosed with autoimmune conditions. Based on organ specificity and possible etiology, autoimmune disorders can be tissue-specific, such as type 1 diabetes (T1D), multiple sclerosis (MS), and autoimmune thyroid disease (AITD); or affect multiple organs, such as systemic lupus erythematosus (SLE), Sjogren syndrome, and rheumatoid arthritis (RA) (25).

**Figure 1:**
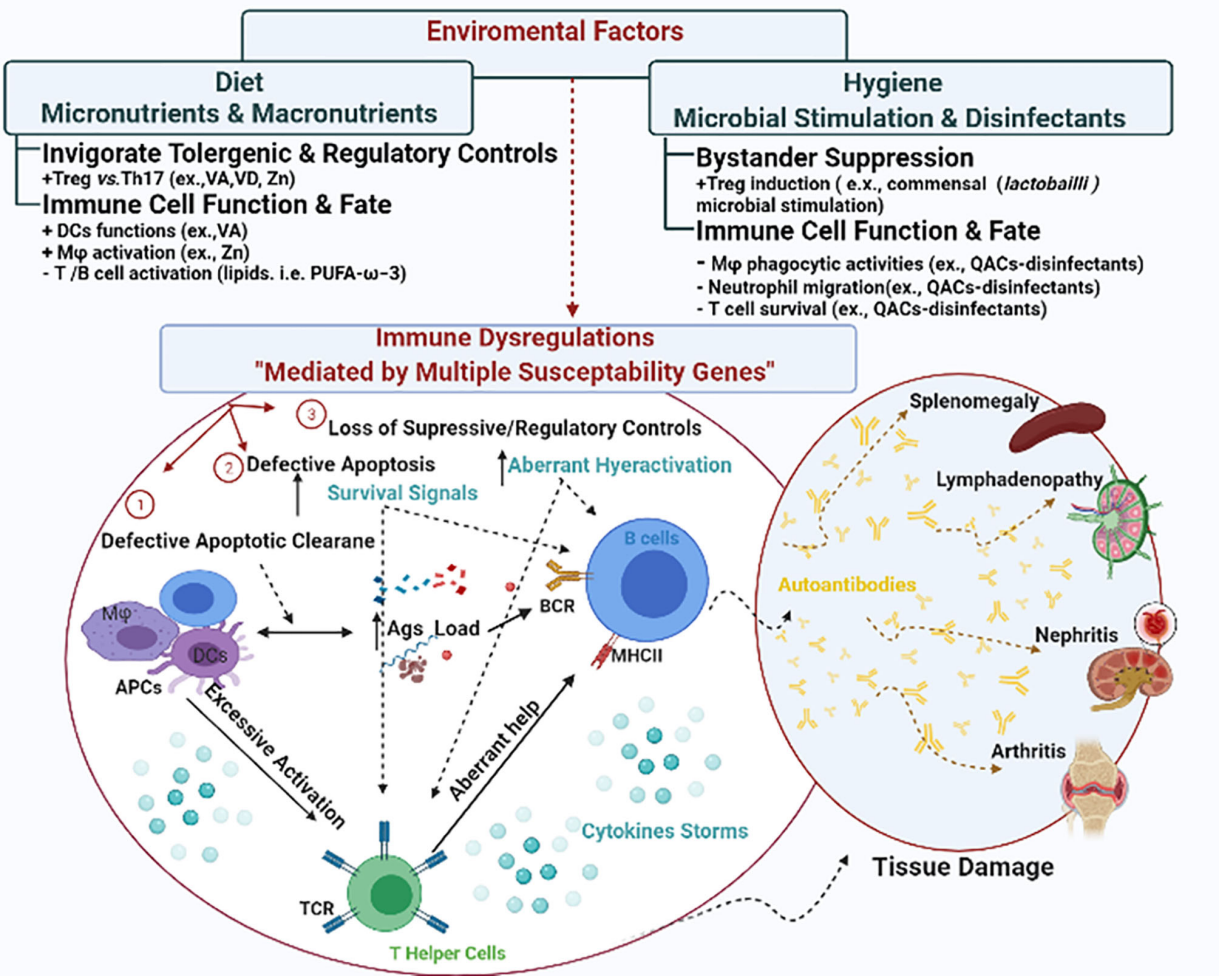
The interplay between environmental factors and genetic susceptibilities in shaping immune dysregulation. Genetic susceptibilities could lead to immune dysregulation *via* different mechanisms including (1) defective apoptotic cell clearance, (2) defective apoptosis, and (3) loss of suppressive regulatory controls. Collectively they lead to disrupted cytokine signals and hyperactivation of autoreactive T and B cells, invigorating a series of tissue damage in various manifestations. In SLE, the manifestations could be presented as splenomegaly, lymphadenopathy, nephritis and arthritis. Environmental triggers either augment or mitigate these mechanisms to shape the autoimmunity.

Different theories have postulated various mechanisms for autoimmune inflammation including the breakdown of central and/or peripheral tolerance (reviewed elsewhere) (26, 27). Here, we summarize several possible mechanisms of immune dysregulation that could lead to systemic autoimmunity (**Figure 1**). These mechanisms include (1) defective apoptotic cell clearance (28), (2) defective apoptosis (29), and (3) loss of suppressive regulatory controls leading to hyperactivation of autoreactive T and B cells. We choose SLE to illustrate how these interrelated mechanisms of immune dysregulation drive autoimmune pathogenesis leading to tissue damage. SLE is a systemic autoimmune disease with a mortality rate nearly 3 times of that in the general population (30). There is no known cure so far.

Autoimmune disorders arise as a result of the interplay among diverse factors including genetic, epigenetic, and environmental triggers (31).

The link between genetic susceptibilities (or inherited anomalies) and the skewness from homeostasis to autoimmunity has been extensively reviewed (32–37). Notably, genetic susceptibilities are far more complex than a single genetic mutation and commonly manifested as multiple genetic susceptibilities.

Environmental factors can either potentiate or mitigate the effects of susceptibility genes, thereby having direct and indirect impacts on the development of immune tolerance and subsequent disease.

The association between environmental exposure and the development of human autoimmune disorders (38) supports the widely accepted notion that environmental triggers can program immune responses. For instance, early-life exposure to infections, vaccines (39), and dietary components (40) mold the immunological memory, consequently shaping how the immune system responds to exogenous stimuli later in life (41, 42). During the recent decades, the tremendous increase in the prevalence of autoimmune conditions coincides with evolving dietary and hygiene styles in Westernized societies (43), indicating a strong influence of environmental factors on autoimmunity (44–49). This is particularly important during pandemic eras since the emergence of unprecedented infections such as COVID-19 is thought to predominate among immunocompromised individuals such as those with preexisting autoimmune conditions who are also at an increased risk of COVID-19 related hospitalization (50). Indeed, autoantibodies against self-proteins such as type 1 interferons (IFNs) were found in cases of severe COVID infection and may account for COVID-19 deaths (51, 52). Interestingly, a recent report has found an increased pre-existing prevalence of anti-IFNα autoantibodies in SLE patients with COVID-19 infection compared to SLE patients without COVID-19 (53), suggesting that these autoantibodies might predispose SLE patients to contract COVID-19. Additionally, a recent study has shown that the activities of different exoproteome-directed autoantibodies (i.e., autoantibodies against immunomodulatory proteins such as cytokines, chemokines, complement components, and cell surface proteins) are dramatically increased in COVID-19 patients compared to uninfected controls (54). In parallel, murine surrogates of these autoantibodies have been shown to exacerbate disease severity in a mouse model of SARS-CoV-2 infection (54), suggesting autoimmunity as a driving factor for severe COVID-19. Together, these studies indicate that pre-existing autoimmunity put autoimmune patients at a higher risk for more severe COVID-19 infection and subsequent mortalities.

Conversely, it is believed that autoimmunity could be the comet tail following COVID-19 infection (54–56) especially in genetically predisposed individuals (57). Therefore, it is crucial to understand the influence of environmental factors on autoimmune regulation to better protect the more vulnerable populations during the COVID-19 pandemic. Specifically, this pandemic has witnessed some unprecedented shifts in human dietary habits both positively (e.g., increased consumption of domestically cooked foods, increased shares of plant-based diets) and negatively (e.g., increased consumption of comfort food) (58). At the same time, people are advised to practice stricter hygiene measures (59, 60). Together these shifts in dietary and hygienic practices could on their own modulate our immune responses and/or autoimmune development or progression. In this review, we propose diet and hygiene as environmental regulators of immunity and discuss their influence on autoimmune development as depicted in **Figure 1**.

### Diet as an Environmental Factor

Dietary practices can influence immune tolerance and disease. Indeed, dietary components, including micronutrients and macronutrients, can affect both innate physical defenses such as epithelial barrier integrity (61–64), antimicrobial peptides (65), and pro/anti-inflammatory cytokines (66–68), as well as adaptive immune cell functionality (69–72). The high incidence of immune-mediated diseases such as autoimmune and allergic disorders in the Western world, where common themes of dietary behaviors exist including increased caloric (fat and carbohydrate) intake with much less fibers and imbalanced dietary fatty acid consumption, pinpoint the immunomodulatory capacities of these macronutrients and their potential causal implications on autoimmune development (47). In contrast, dietary patterns that are mostly plant-based such as Mediterranean or DASH diets have been shown to contain anti-inflammatory and antioxidant components (73, 74) that could impose protective effects against autoimmunity (75). Among these, plant-derived phytochemicals including polyphenols (76) such as flavonoids (77–79) and isoflavones (80, 81) have been extensively investigated for healthful modulation of autoimmunity.

To this end, the upside of the COVID-19 pandemic is its impact on the unprecedented dietary shifts to less processed and more plant-based dietary sources (82). Interestingly, plant-based diets are associated with lower odds of moderate-to-severe COVID-19 and may provide protective support against severe COVID-19 infection (83). Indeed, such dietary trends with COVID-19 emergence can not only benefit to restrain COVID-19 infection in normal people but also be supportive strategies for the vulnerable populations with autoimmune conditions. For example, quercetin is a natural flavonoid derived from different plant sources that has various anti-inflammatory and antioxidant immunomodulatory capacities (84, 85). Based on pharmacology and molecular docking, quercetin has been proposed as a potential protective treatment against acute renal injury, one of the most serious complications reported in hospitalized patients with COVID-19 infection (86–88). At the same time, quercetin has been shown to attenuate various autoimmune pathologies in human and murine rheumatoid arthritis (RA) (89–92), and in experimental models of inflammatory bowel diseases (IBD) (93, 94) and SLE (95). In the following section, we review how different micronutrients and macronutrients modulate immune health and autoimmune outcomes and discuss the potential advantageous roles of dietary manipulation in mitigating the risks of COVID-19 infection in autoimmune patients.

## Micronutrients and Autoimmunity

Micronutrients including vitamins (such as vitamins E, A, and D) and minerals (such as selenium, copper, and zinc) are long known to possess capabilities to modulate immune responses. Micronutrients can tone every aspect of both innate and adaptive responses (96). In the following section, we will discuss the role of vitamins and minerals in maintaining health and how their deficiencies contribute to autoimmune diseases.

### Vitamins

Vitamins have important functions in maintaining immune health through their antioxidant capacities (97). There is a huge body of literature supporting the notion that oxidative stress plays a crucial role in autoimmune pathogenesis (98–100). For instance, oxidative degradation of lipids that occur in the cellular membrane in a process known as lipid peroxidation is mediated by free radicals (101, 102), which could impair the integrity of the cell membrane (103), induce cellular death (101) and accumulate apoptotic products, subsequently initiating autoimmunity (104–106). In addition, intracellular oxidative signaling could increase the responsiveness of autoreactive immune cells such as T lymphocytes and drive their autoimmune pathogenicity (107). Moreover, the extracellular release of oxidative products including reactive oxygen species (ROS) and proteases by innate immune cells such as neutrophils could initiate autoreactivity and lead to collateral tissue damage (108). Interestingly, oxidative stress has also been proposed to be associated with COVID-19 pathogenesis (109–111). Therefore, antioxidant vitamins could provide potentially beneficial supportive care for autoimmune patients with COVID-19. Here, we review the immunomodulatory functions of vitamins E, A, and D in autoimmunity and discuss their potential implications in COVID-19.

Vitamin E (VE) as an antioxidant can diminish the secretion of ROS (e.g., by monocytes) (112), thereby protecting against oxidative cell stress and indirectly maintaining immune cell functionality. Therefore, it has been suggested that the relative deficiency of VE could initiate autoimmunity (113). Although data from human studies are not conclusive—where some reports showed that VE levels were increased in patients with systemic autoimmunity of primary Sjögren’s syndrome (114), while others correlated VE deficiency with the development of neurological complications of celiac disease (115) or showed no difference in VE levels in patients with IBD (116)—murine studies more decisively support the potential beneficial effect of VE in the treatment of autoimmune conditions such as RA (117, 118). Importantly, based on data from murine lupus, VE is proposed as a safe treatment option for SLE (119, 120) where the supplementation reduces hallmarks of SLE such as the levels of anti-double-stranded DNA (anti-dsDNA) IgG antibodies (121) and counteracts oxidative stress (and lipid peroxidation) that would otherwise contribute to more debilitating SLE manifestations (122, 123). In human studies, VE supplementation as part of a treatment regime improved the oxidative and nitrosative biomarkers and disease activity in SLE patients (124).

**Figure 2:**
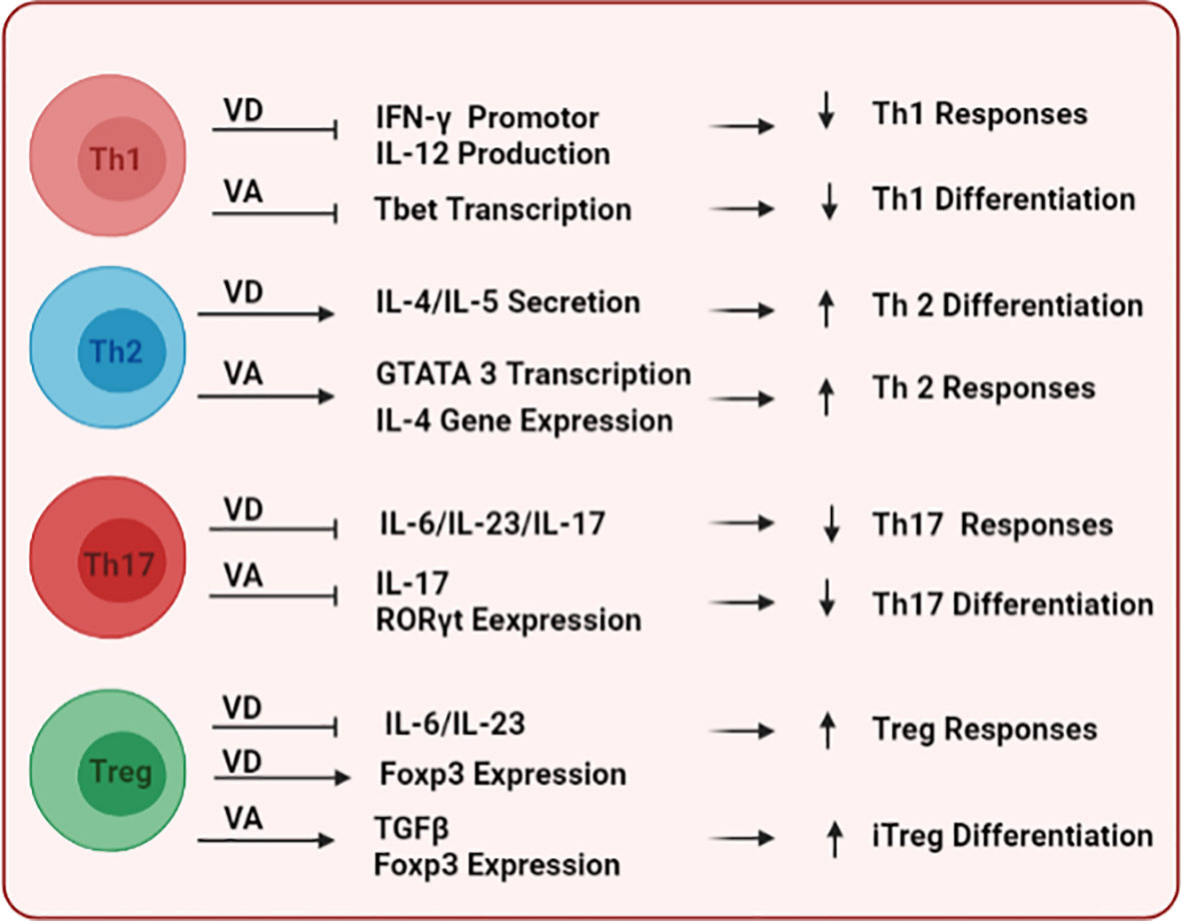
The immunomodulatory effects of vitamins D and A on T cells. Following their binding to DNA responsive elements of target genes, both vitamins differentially modulate key transcription factors and stimulatory cytokine signals to shape the commitment and functional responses of multiple T-cell subsets including Th1, Th2, Th17, and Treg.

Vitamins A and D (VA and VD, respectively) are known to modulate the differentiation of immune cells, their tissue homing, and effector functions. On the innate arm, VD and VA have context/tissue-dependent functions that may antagonize each other. VD has been shown to mitigate the maturation of DCs, inhibiting their expression of maturation markers MHCII and CD80/86 (125), as well as reducing their production of the pro-inflammatory cytokine IL-12 (126), collectively inducing the tolerogenic phenotype of DCs (127, 128). In contrast, VA has context-dependent effects on DCs. Although VA is a key player in the induction of mucosal tolerance (129), it can also enhance DC maturation and their antigen-presenting capacities in the presence of pro-inflammatory stimuli (130). In addition, VA can increase the expression of matrix metalloproteinase-9 (MMP9) to enhance the migration of inflammatory DCs to lymph nodes (131). Together, these immunomodulatory capacities of VD and VA could represent a tango for *in-vivo* tuning of DC functions. Importantly, since DCs could modulate self-tolerance and the development of autoimmune disease such as SLE as we previously reviewed (132), VD (133)-, and VA (134, 135)-mediated regulation of DC function could indirectly impact the development of tolerance and disease. On the adaptive arm, both VD and VA can affect the differentiation of T cell subsets, including T helper (Th)1 (proinflammatory) *vs.* Th2 (anti-inflammatory), and T regulatory (Treg; immunosuppressive) *vs*. Th17 (proinflammatory) as depicted in **Figure 2**. For example, VD (67, 125, 136–138) and VA (139–141) have been shown to inhibit Th1 differentiation and responses while supporting the development and responses of Th2 lymphocytes. In addition, both VD (142, 143) and VA (144–146) potentiate the development of Treg over Th17. Importantly, the imbalance between these different T cell subsets is a driving force for autoimmune development (147, 148).

Ecological associations between low VD levels and the incidence of different autoimmune conditions such as IBD, MS, T1D, and RA have been reported in areas with limited sun exposure (149–151). It is widely hypothesized that deficiency of VD is associated with the development of different autoimmune conditions where polymorphism in VD receptors (VDR) are implicated among the causal risks for autoimmune conditions including AITD (152, 153), T1D (154), MS (155), RA (156) and SLE (157). In addition, VD deficiency is associated with more aggravated autoimmune flares in SLE (158–161) by promoting memory B cells responses (162) and subsequent autoantibody production (163). These observations strengthen the potential use of VD supplementation to control autoimmune disease (164). Importantly, plasma (165) and serum (166) levels of VD have been shown to be depleted in COVID-19 patients. Additionally, low levels of VD are also correlated with the severity of COVID-19 progression in those patients (166). Meanwhile, VD supplementation during or just before COVID-19 contraction can mitigate disease progression and enhance the survival rate in infected patients (167). Together these reports support the potential healthful effect of VD in autoimmune patients to combat COVID-19 severity and fatality.

In contrast, it remains to be determined if VA deficiency or polymorphisms in VA receptors are causally associated with autoimmune development. Although serum retinol levels have been negatively correlated with MS activity (168), and hypovitaminosis A has been detected proceeding the clinical diagnosis of SLE (169, 170), it is still to be elucidated whether VA deficiency is a driving factor for autoimmunity. Due to their immunomodulatory capacities, retinoids have been proposed as potentially beneficial adjuvants controlling autoimmune disorders (171). The most active metabolite of VA, all-*trans*-retinoic acid (tRA), has been shown to control pathogenic T cell subsets such as IL-17 secreting γδ T cells and sufficiently ameliorate experimental autoimmune encephalitis (EAE), a murine model of MS. Similarly, we and others have shown the protective effects of VA on murine SLE especially during the active stage of the disease (172–174). In future investigations, we propose to (1) investigate the potentially detrimental effects of VA deficiency on the progression of renal inflammation in genetically-prone SLE, and (2) delineate the molecular and transcriptional mechanisms by which hypovitaminosis A promotes systemic autoimmunity. Importantly, a recent study conducted in hospitalized COVID-19 patients with respiratory failure showed that serum retinol levels were significantly lower compared to healthy controls (175). This report’s findings support the idea that COVID-19 infection is associated with retinoic acid depletion syndrome (176) and that VA supplementation might be a beneficial approach especially in areas of limited medical resources (177). Therefore, considering its complex roles in modulating autoimmunity, VA might be a crucial element to be considered for monitoring and supplementation as needed for autoimmune patients with COVID-19 infection.

It is important to note that for clinical applications, optimal levels of these vitamins as combinations may be crucial for achieving the harmony of their action. Future investigations on the vitamins’ modulation of autoimmunity as an overlapping circuit rather than solo-induced effects will shed more light on their therapeutic potential in clinical settings.

### Minerals

Minerals, or specifically trace elements such as selenium, copper, and zinc, modulate immune functions in various indirect ways. Generally, minerals can have antioxidant capacities and consequently maintain the structural integrity of essential biological molecules (e.g., maintaining cell membrane stability) (178), participate in enzymatic activities (179), and potentiate energy production and use during cellular metabolism (180). Importantly, some trace elements are needed to facilitate cell signaling (181, 182), where their binding to target ligands/receptors regulates essential processes including gene expression and protein synthesis (183, 184). Selenium, for example, acts as an antioxidant, eliminating free radicals and subsequently maintaining immune system functions (185). It also has catalytic activities and is essential for the enzymatic activity of glutathione peroxidase to inhibit lipid peroxidation (186). This is particularly important for protecting cell membrane phospholipids, consequently maintaining cell membrane structure and preventing oxidative mitochondrial damage in immune cells such as macrophages (187–189). Therefore, selenium deficiency is associated with increased inflammation (190, 191) and autoimmunity as in celiac disease and AITD (192). Similarly, meta-analysis of genome-wide association studies (GWAS) have predicted high selenium levels to be associated with a decreased risk for SLE (193). Indeed, selenium supplementation in murine models of SLE has been shown to improve survival (194) and mitigate various hallmarks of the disease including splenomegaly and autoantibody production (195). In addition, a recent cohort study has shown that severe COVID-19 patients exhibit a pronounced deficit in total serum selenium and selenoprotein levels, and that selenium status is significantly higher in COVID survivors compared to non-survivors, suggesting the therapeutic potential of selenium supplementation in severely diseased and/or selenium-deficient COVID-19 patients (196).

Aside from these non-specific immunomodulatory capacities, minerals can directly modulate both innate and adaptive immune cell activities and be involved in the activation of key signaling molecules such as NF-κB. Zinc, for example, can be both pro- and anti-inflammatory and plays an essential role in maintaining both innate and adaptive cellular responses (197). Zinc promotes NF-κB signaling and subsequently the production of pro-inflammatory cytokines from macrophages (198, 199). The utilization of zinc by macrophages enhances their phagocytic activities (200, 201). Therefore, lower circulatory levels of zinc could be linked to macrophage-related autoimmune diseases such as RA (202). Meanwhile, zinc can also induce tolerogenic DCs in both *in-vivo* and *in-vitro* settings (203). Thus, zinc deficiency could compromise the interface between innate and adaptive immunity leading to skewing of Th cell balance (203). Zinc is essential for T lymphocyte proliferation (204) and activation (205) through promoting IL-2 signaling. It also promotes the upregulation of IL-12 signaling and Th1 transcription regulator T-bet (206). Consistently, zinc deficiency reduces the levels of Th1-polarizing cytokines (e.g., IFNγ) (207). Therefore, a shift of Th1/Th2 balance to Th2 has been linked to zinc deficiency. However, zinc can enhance the differentiation of Treg cells through upregulating FOXP3, where it increases the phosphorylation of Smad proteins, allowing for its binding to the *Foxp3* promoter (208). Furthermore, zinc can suppress Th17 differentiation through inhibiting signal transducer and activator of transcription 3 (STAT3) signaling (209) as well as memory Th17 responses *via* inhibition of the IL-1β/IL‐1 receptor‐associated kinase 4 (IRAK4) phosphorylation (210). Thus, zinc can benefit by tuning T cell-driven autoimmunity as shown in EAE (211, 212). In human studies, lower systemic zinc levels have been reported in different autoimmune conditions (213) including MS (214) and SLE (215, 216). Interestingly, COVID-19 patients also have lower zinc levels that are correlated with disease complications and prolonged hospital stays (217). Together, these studies warrant further investigation on the immunomodulatory capacities of zinc in autoimmune and/or other high-risk patients for COVID-19.

In summary, micronutrients are essential for fine-tuning the development and function of immune cells. Altered homeostasis of micronutrients as seen in various autoimmune diseases could critically influence immunity and promote autoimmune dysregulations.

It is noteworthy that although evidence-based findings are still being uncovered on the causal relationship between micronutrient deficiencies and more severe COVID-19, as well as the potential healthful effects of micronutrients on COVID convalescence, the immunomodulatory functions of micronutrients may support their roles in combating COVID-19 infection (218–220). Therefore, for autoimmune patients in the COVID-19 pandemic, the potential benefits of micronutrients as discussed above urge for regular monitoring of their levels and if deemed necessary, supplementations in individuals with specific micronutrient deficiencies.

## Macronutrients and Autoimmunity

Macronutrients have diverse immunomodulatory functions that dynamically modulate immune cell responses to shape autoimmune outcomes. Data exploring the implication of specific macronutrients in COVID-19 are lacking. However, it is well established that overconsumption of energy-yielding macronutrients could lead to obesity (221), a condition considered as one of the major risk factors of severe COVID-19 infection (222–225). Here, we review current knowledge on the modulation of autoimmunity by different macronutrients and discuss points to be considered with regards to COVID-19 infection in autoimmune patients.

### Carbohydrate, Fat, Protein

Dietary carbohydrates are usually in the form of polysaccharides, oligosaccharides (e.g., fructo-oligosaccharides), disaccharides (e.g., lactose), and monosaccharides (e.g., glucose and fructose) (226). Carbohydrates, especially non-digestible polysaccharides and oligosaccharides (e.g., in prebiotics), exert both direct and indirect immunomodulatory capacities. Indirectly, they act as a source of energy for gut microbiota which, in turn, could modulate immune responses *via* different mechanisms as previously reviewed (227, 228). Directly, they could modulate both innate and adaptive effector immune functions involving epithelial tight junctions, cytokine/chemokines, and antibody production as reviewed elsewhere (229). Indeed, dietary polysaccharides are sensed by different immune cell receptors including complement receptor 3 (CR3), Toll-like receptor (TLR) as well as dectins (carbohydrate-binding proteins) (230); subsequently, they act as immunogens to stimulate immune responses. Notably, plant polysaccharides can be the interface linking innate and adaptive immune cell activation. They primarily stimulate the proliferation and activation of innate cellular components such as natural killer (NK) cells (enhancing their cytotoxic activities) and macrophages (augmenting their production of TNFα and IL-6 as well as their lysosomal activities and nitric oxide production) (231). In turn, these innate components activate their adaptive counterparts, inducing Th cell differentiation and antibody production from B cells as previously detailed (231).

The effector functions of immune cells rely primarily on glucose (232). Therefore, it is not surprising that high carbohydrate intake could exacerbate the hyperactivation of immune cells in chronic inflammatory and autoimmune conditions. Indeed, modern diets containing high levels of processed fructose-rich carbohydrates increase the incidence of chronic inflammation (233, 234). High glucose intake augments ROS-dependent activation of TGFβ and consequently the induction of Th17, thereby exacerbating autoimmune conditions as reported in murine models of MS (i.e., EAE) and colitis (235). In addition, excessive carbohydrate intake increases the levels of circulatory inflammatory markers such as IL-6 and C-reactive proteins (CRP) in SLE patients (236). In contrast, natural glucose derived from plant-based carbohydrates such as cell wall-based cellulose (which is an insoluble fiber and composed of unbranched β-1,4-linked glucose monomers) has been shown to reduce the frequency and number of pro-inflammatory T cells and promote autoimmune suppressive Th2 responses in EAE (237). These findings illustrate the pivotal effects of dietary carbohydrates on immune cell functions and warrant further investigations towards more mechanistic insights with a specific emphasis on autoimmune modulation.

It is also crucial to maintain a balance between carbohydrates and other macronutrients including dietary fats and proteins. Although plant-based lipids (e.g., phytosterol from vegetable oils) could beneficially modulate autoimmune inflammation as reported in EAE (238, 239), it is controversial how fat-modulated diets precisely shift immune responses and subsequently autoimmune progression. If only the level of fat consumption is changed, reduced dietary fat levels increase the proliferation of human peripheral blood mononuclear cells (PBMCs) following both mitogens and inflammatory cytokine stimuli (240), suggesting better responses to pathogenic invaders (241). However, recent studies propose that ketogenic diets of low carbohydrate and high fat intake potentiate more protective γδ T cell responses against infectious triggers (242). This emphasizes the importance of considering the levels of all macronutrients together when assessing immune modulation under steady-state conditions.

Under autoimmune conditions, on the other hand, a large pool of evidence proposes that a high-fat diet (HFD) adversely impacts the immunopathogenesis of different autoimmune diseases as widely investigated in SLE (243). For example, high fat intake resulted in defective phagocytic and cytotoxic activities of macrophages and NK cells, respectively, and this was associated with earlier onset and exacerbated autoimmunity in lupus-prone NZB/W F1 mice (244). In addition, HFD further increased TLR7 expression and signaling in TLR8 knockout mice (a spontaneous murine lupus model characterized by increased TLR7 signaling) leading to exacerbated kidney inflammation due to increased production and renal deposition of autoantibodies (245). Furthermore, HFD-induced obesity increased the T follicular helper (Tfh) cell activity in MRL/lpr mice and consequently the activation of B cells, exacerbating SLE-associated splenomegaly and potentiating IgG production and their glomerular deposition (246). In contrast, a low-fat, isoenergetic diet designed to provide no more than 20% energy from fat a day, together with supplementation of 1 g of fish oil, effectively attenuated disease activity in SLE patients (247). Although it is yet to be tested whether high fat/low carbohydrate ketogenic diets are beneficial in SLE patients, a potential positive effect of ketogenic diets has been reported for some autoimmune patients with MS (248) or T1D (249). Interestingly, a ketogenic diet may also provide supportive care for COVID-19 patients (250), reducing their need for the intensive care unit (251). This again emphasizes the importance of considering the levels of all macronutrients together on immune modulation under autoimmune status and highlights the importance of personalized care plans for autoimmune individuals with COVID-19 infection.

Not only the quantity but also the type of consumed fat could differentially affect the immunopathogenesis of autoimmunity. Dietary fats could be in the form of saturated fat, *trans*-fat and unsaturated fat. Increased intake of saturated fat accelerates the disease relapse in children with MS (252). Increased consumption of *trans*-fat increases the levels of free radicals that damage the integrity of immune cell membranes and contribute to autoimmunity (243). In contrast, the incorporation of Mediterranean diets that significantly increases the ratio of unsaturated to saturated fats has been linked to improved autoimmune pathologies (e.g., alleviating RA symptoms) (253). Notably, a diet rich in polyunsaturated fatty acids (PUFA) can improve the overall clinical scores and elicit anti-inflammatory effects in SLE (254). Similarly, a recent report has shown a negative correlation between adherence to Mediterranean diets and COVID-19 cases in Spain and other countries (255), supporting a potential protective role of PUFA-rich Mediterranean diets against COVID-19 (256, 257).

Indeed, supplementation of PUFA such as omega-3 (ω−3) augment the activities of antioxidant enzymes and diminish autoimmunity (243). Several studies have shown that ω−3 enriched diets dramatically reduce SLE-associated inflammatory markers, lupus progression (mitigating glomerulonephritis) and improve survival in different mouse models of SLE (258–262). This is achieved mostly through potentiating the effects of antioxidant enzymes (such as superoxide dismutase and glutathione peroxidase) which, in turn, potentiate the ability of renal cells to eliminate harmful free radicals (e.g., reactive oxygen intermediates) (259, 261). Moreover, a diet rich in ω−3 (e.g., with fish oil) reduces the level of anti-dsDNA antibodies (258, 261), circulating immune complexes, and their renal deposition (258). Furthermore, fish oil can reduce the expression of renal pro-inflammatory cytokines including IFNγ, IL-12, TNFα (259, 261), and renal profibrotic molecules such as TGFβ and fibronectin-1 (261). By downregulating these molecules, fish oil diminishes the age-associated activation of NF-κB signaling in lupus mice, thereby mitigating lupus nephritis (259). Indeed, fish oil is a dietary source for ω−3 PUFA that exerts anti-inflammatory capacities through dampening immune cell responsiveness to IFNγ (263). Recent findings have shown that ω−3 PUFA could also downregulate biomarkers associated with T and B cell activation and/or differentiation and leukocyte recruitment such as CD80, CTLA-4, IL-18, CCL5, CXCR3, IL-6, TNFα (264). Similarly, they mitigate inflammatory markers associated with intestinal inflammation/colitis such as IL-6, inducible nitric oxide synthase (iNOS), cyclooxygenase-2 (COX-2), and leukotriene B_4_ (LTB_4_) (265). Collectively, these beneficial actions of ω-3 PUFA on autoimmune development (especially SLE) could be targeted in future human intervention studies. Interestingly, for their immunomodulatory capacities, ω−3 PUFA have been recently proposed as part of the supportive care for COVID-19 patients (266, 267), as recent pilot data suggest that a higher ω-3 index may lower the risk of COVID-19 fatality (268). Therefore, diets rich in PUFA might confer benefits for autoimmune patients with SARS-CoV-2 infection.

Dietary proteins are the third component of macronutrients in a balanced diet and are hydrolyzed in the gut to generate amino acids and peptides. Proteins can, directly and indirectly, modulate immune functions. Directly, dietary proteins-derived amino acids can serve as major energy sources for leukocytes, enhance the development of immune cells from hematopoietic progenitors, and modulate their effector functions as previously reviewed (269). Indirectly, proteins are utilized through gut microbiota which in turn mediates the interplay between protein metabolites and the host immune system as detailed elsewhere (270). Indeed, exposure to dietary protein antigens is necessary for the maturation of the immune system (271, 272). Feeding of weanling mice a protein-deficient diet that had only free amino acids resulted in a poorly developed immunological profile that resembled that of newborn or germ-free (GF) mice (271, 273), which is characterized by reduced secretory IgA and systemic Ig levels as well as less-developed gut-associated lymphoid tissues (271, 273). Moreover, in addition to playing an integral role in the development of oral tolerance (273, 274), early-life intake of protein antigens is crucial for Th1 differentiation (271), B cell responses, and class switching (275), thereby helping to augment the immunity against infectious invaders later in life (272). Together, these studies suggest that dietary protein malnutrition (PM) could adversely impact immunity. Interestingly, both PM and excessively high protein intake could exacerbate inflammation. PM could potentiate intestinal mucosal damage following inflammatory stimulation (e.g., zymosan-induced systemic inflammation), allowing bacterial translocation and gut-induced septicemia (276). In parallel, in contrast to proteins from plant origins that could mitigate inflammation (277), a high-protein diet, especially from animal origin, aggravates both acute and chronic dextran sulfate sodium (DSS)-induced colitis *via* promoting the pro-inflammatory responses of macrophages (278). Furthermore, high protein intake has been associated with deterioration of renal disease involving glomerular hyperfiltration (279). Meanwhile, a low-protein diet could help the management of chronic kidney diseases (280). As kidney inflammation, namely lupus nephritis is the most life-threatening manifestation of SLE, modulation of protein intake could be of great importance. Indeed, moderate dietary protein intake of 0.6 g/kg daily improves renal functions in SLE patients (281). Restriction of protein intake in NZB/W F1 mice improved the disease outcome, inhibited splenomegaly, and maintained immune cell responsiveness to mitogenic stimulation (282).

Together, these observations suggest that dietary protein modulation can influence immune components in the pathogenesis of autoimmune disorders such as SLE, therefore warranting further studies to elucidate the underlying cellular and molecular mechanisms.

### Energy Intake

Epidemiological association between obesity and autoinflammatory disorders (283) suggests that excessive caloric/energy intake could lead to the breakdown of immunological tolerance. Excess caloric intake in early life increases the incidence of IBD (284) and autoimmune thyroiditis (285) in adulthood. In contrast, caloric/energy intake restriction (EIR) can improve the autoimmune/inflammatory outcomes as have been reported in experimental models of SLE (286–291), EAE (292–295), and Sjogren’s syndrome (296).

Immune responses are high energy-dependent to fuel the biosynthetic needs of activated immune cells. The bioenergetic demand of immune cells is achieved *via* three interconnected metabolic pathways including oxidative phosphorylation (which occurs in the mitochondria of naïve cells), glycolysis (which occurs in the cytoplasm of activated and proliferating cells), and tricarboxylic acid (TCA) cycle (which occurs in the mitochondria of activated and proliferating cells) (232). The immune system is dynamically active with continuous changes in cellular activities such as those between resting naïve cells and proliferating effector cells, and those of memory/long-lived cell populations that might be quiescent but ready to undergo rapid proliferation upon stimulation. Therefore, the cellular energy demand is variable based on the phases and activities of immune cells (297–299). For instance, for a cell in a resting state (e.g., naïve T or B cells), the need for energy will be towards maintaining its minimal metabolic and biosynthetic requirements that are directed for building cellular components. Therefore, the cellular energy expenditure is met through oxidative phosphorylation and targets preserving cellular integrity. In contrast, effector immune cells undergo rapid proliferation and have a wide range of effector functions as represented in the production of effector molecules such as cytokines, chemokines, inflammatory mediators, and immunoglobulins. This will require upregulation of fuel uptake and aerobic glycolysis to satisfy higher metabolic configurations needed for both bioenergetic and biosynthetic pathways (297–299). Importantly, these shifts of cellular energy phases could be largely dependent on nutrient and energy supplies in the microenvironment. Moreover, energy/caloric intake can influence cellular metabolism as well as phenotypic and functional capacities of immune cells (300–302). For instance, due to chronic metabolic stress leading to ATP depletion, chronically activated T cells in SLE patients fulfill their need for ATP through oxidative phosphorylation rather than upregulating the aerobic glycolysis (303). In parallel, continuous low-grade inflammation due to excess energy intake (as in obesity) exacerbates the proinflammatory phenotype of different immune cells, favoring the differentiation of Th1 and Th17 subsets while diminishing the frequency of Treg cells (304), thereby predisposing individuals to autoimmunity (283).

Furthermore, energy intake can shape the fate of immune cells (e.g., differentiation and effector functions) through modulating key signaling pathways and transcription factors which in turn contribute to autoimmune pathogenesis. For instance, activation of T cells through TCR signaling and CD28 co-stimulation leads to activation of the phosphatidylinositol-4,5-bisphosphate 3-kinase (PI3K)/Akt pathway (305–307), which subsequently turns on the mammalian target of rapamycin (mTOR) pathway, where mTOR is of two functional complexes, mTORC1 and mTORC2 (308). mTOR is a central regulator for T cell differentiation and homeostasis (309, 310). Under normal nutrient and energy conditions, mTOR (especially mTORC1) senses the microenvironment and upregulates the expression of glycolytic genes (311), allowing for biosynthetic anabolic processes needed for cell proliferation (309). However, excessive functions of mTOR can disrupt cellular differentiation and predispose individuals to autoimmune conditions such as RA (312) and SLE (313). Augmented mTOR activities under autoimmune conditions are not limited to T cells, where mTORC1 expands pathogenic T cell populations including Th17 and the CD4^-^CD8^-^ double-negative T (DN-T cells) while suppressing Treg cells (313); but also B cells (314–316), DCs and macrophages (317, 318). Activation of mTORC1 precedes the onset of SLE as a result of chronic metabolic stress with long-term ATP depletion and mitochondrial hyperpolarization (319, 320). In contrast, during energy restriction, rapid inhibition of mTOR occurs, allowing the shift to catabolic processes to maintain the energy required for cell survival (321, 322); at the same time, Treg cells become responsive to TCR stimulation, thus enhancing their proliferation (323). Therefore, optimizing the cellular metabolic shifts could open new therapeutic avenues for the treatment of autoimmunity (324, 325).

Consistent with this notion, EIR can suppress the immunopathogenic responses associated with autoimmunity and chronic inflammation including hyperactivation of cellular and humoral responses (326). For instance, EIR reduces antigen processing by macrophages as well as the T cell-dependent activation of B cells (326). Similarly, EIR diminishes the inflammatory activities of circulating monocytes and their mobilization from the bone marrow without compromising their emergency egression during acute infection and tissue repair (327). Through these mechanisms, EIR has been shown to modify the autoimmune/inflammatory pathogenesis of various disorders (286–296). For example, EIR can dampen the SLE progression. Early dietary modulation through caloric restriction by the time of weaning reduces SLE-associated lymphadenopathy in mice (328). Caloric restriction significantly reduces B cell frequencies and their activation (291), reduces circulatory anti-dsDNA antibodies, and prevents the increase of possibly pathogenic T cell subsets such as DN-T cells (329), while maintaining a higher percentage of naïve T cell subsets (291) as well as the responsiveness of lymphocytes to mitogenic stimulation (329). In addition, lupus-prone NZB mice with chronic energy/calorie intake restriction (CEIR; fed a 40% caloric reduction diet that was relatively low in fat and high in carbohydrates) prolongs the survival and delays the disease initiation (286). In NZB mice as well as other lupus-prone murine models including MRL/*lpr* and BXSB, CEIR diminishes proliferation of lymphoid cells in the spleen, thymus, and mesenteric lymph node (MLN) (287), downregulates the transcript levels of proinflammatory cytokines such as IFNγ and IL-12, reduces IgA and IgG2a autoantibodies (288), and decreases molecules associated with fibrinogenesis such as platelet-derived growth factor (PDGF) (289), subsequently ameliorates lupus-associated kidney inflammation or glomerulonephritis. These observations emphasize the potential use of EIR in the treatment of SLE.

In summary, calorie restriction (e.g., intermittent fasting) and fat/carbohydrate modulation (e.g., ketogenic diets) may provide novel opportunities against autoimmunity by regulating both adaptive and innate immune activation pathways (242, 330). However, further studies are warranted to establish the safety and clinical efficacy of immunometabolic treatment strategies in autoimmunity. In parallel, it is essential to ensure that the modulated diets do not lead to malnutrition that promotes immune dysfunction and increases the risk and severity of infections (331). Notably, malnutrition predisposes COVID-19 patients to more severe disease in an age-dependent manner (332) and increases the odds of their fatality (333). Indeed, COVID-19 patients may require higher energy intake and increased protein consumption (334), and it is a necessity to consider the immunomodulatory influence of macronutrients for proper supportive managements of autoimmune patients with COVID-19 infection.

In **Table 1**, we present a short list of several nutrients that have shown beneficial effects in murine models of SLE where human studies are warranted.

**Table 1:** The positive influence of specific dietary nutrients on SLE.

Dietary Factors	Data on murine studies of SLE	Human studies that warrant further investigation	Proposed research directions
**VE**	VE supplementation to NZBWF1 diminished anti-dsDNA autoantibodies and counteracted oxidative stress (121).	Supplementation of VE with prednisolone reduced anti-dsDNA antibodies independently of its antioxidant activity (335). Supplementation of VE with Nigella sativa improved oxidative and nitrosative biomarkers and SLE disease activity favoring antioxidant therapy in SLE (124).	• Delineating the effects of VE independently of other components of treatment regimes. • Exploring the possible disease-stage-dependent or tissue-specific outcomes of VE supplementation.
**VD**	Low levels of VD promoted memory B cells in *Act1^-/-^* mouse (162). VD deficiency increased type 1 IFN gene expression in MRL/lpr mice (336). Treatment of MRL/lpr mice with VDR agonist paricalcitol mitigated lupus nephritis *via* modulating the NF-κB/NLRP3/caspase-1/IL-1β/IL-18 axis and suppressing NF-κB nuclear translocation (337).	A significant negative association between serum VD and memory B cells was confirmed in a cohort of SLE patients (162). SLE patients with high anti-dsDNA autoantibodies (338) or renal involvement (339) are at higher risk for developing hypovitaminosis D; and low levels of VD are correlated with high disease activity (161). Polymorphism in VDR genes has been reported in SLE patients (170).	• Investigating whether VD deficiency is a cause or a sequelae to autoimmune progression. • Reforming genetic association data linking certain genetic susceptibilities and possibilities of VD deficiency for better personalized therapeutic approaches. • Establishing the desired doses for prophylactic and/or therapeutic supplementation of VD for SLE patients. • Testing the potential efficacy and safety of VD supplementation on SLE-associated inflammatory and hemostatic markers in long-term studies.
**VA**	Supplementation of all-*trans*-retinoic acid (tRA) to murine lupus models drove disease-stage dependent effects on the development of lupus nephritis. During the active disease stages in MRL/lpr and pristane-induced lupus mouse models, tRA oral dosing showed beneficial effects on renal inflammation (172–174).	Hypovitaminosis A has been detected preceding the clinical diagnosis of SLE (169).	• Investigating whether VA deficiency is a driving factor for SLE progression. • Genome-wide association studies to establish whether polymorphisms in VA receptors are causally associated with SLE development. • Determining the desired doses of prophylactic and/or therapeutic supplementation of VA. • Testing the safety and efficacy of VA supplementation in SLE patients with different disease manifestations.
**Se**	Se supplementation improved the survival in NZB/NZW F1 mice (194). SE treatment attenuated SLE-associated splenomegaly in B6.Sle1b mice (195). SE supplementation in B6.Sle1b mice significantly reduced total and germinal center B cell numbers, and anti-dsDNA and anti-SmRNP autoantibodies (195).	Meta-analysis of genome-wide association studies predicted high Se levels to associate with a decreased risk for SLE (193). Circulatory Se levels are lower in patients with SLE compared to age- and sex-matched healthy controls (216).	• Exploring the potential therapeutic effect of Se supplementation for SLE patients in large-scale studies. • Elucidating the underlying mechanisms of the potential protective role of Se on the risk for SLE.
**ω−3 PUFA**	ω−3 enriched diets dramatically reduced lupus progression, mitigating glomerulonephritis and improving survival in different mouse models of SLE (258–261). ω−3 diminished anti-dsDNA antibodies (258, 261), and circulating immune complexes and their renal deposition (258). ω−3 potentiated the effects of antioxidant enzymes, enhancing the ability of renal cells to eliminate harmful free radicals (259, 261). ω−3 reduced the expression of renal pro-inflammatory cytokines including IFNγ, IL-12, TNFα (259, 261), and renal profibrotic molecules such as TGFβ and fibronectin-1 (261).	Fish oil (the marine source of ω−3) together with a low-fat diet significantly modified SLE activity (247). A population-based study suggested that a higher dietary intake of ω−3 fatty acids and lower ω−6: ω−3 ratios were positively associated with patient-reported favorable outcomes of SLE activity index (340).	• Conducting longer-term trials with larger patient sample sizes to establish the long-term outcomes of ω−3 PUFA supplementation on the SLE activity index. • Determining the therapeutic efficacy and safety of ω−3 PUFA as part of the therapeutic regimes that include other immunosuppressive agents.

## Hygiene as an Environment Factor

According to the hygiene hypothesis, microbial stimulation might be particularly crucial for immune education during early life, leading to less self-reactivity, thereby mitigating the development of autoimmunity later in life. This might be conceivable since both neonatal innate and adaptive defenses are biased towards maintaining tolerance developed *in utero* (as reviewed elsewhere) (341, 342). For instance, neonatal APCs have reduced antigen-presenting and costimulatory capacities (343, 344). Similarly, neonatal T cells have a default programming toward a Treg phenotype (345) and exhibit more bias toward Th2 cell responses (346). Together, these evidences could explain the increased susceptibility to infections and higher disease morbidity in neonates (347). Interestingly, challenging the neonatal defenses with infections reshapes their immune responses (348) and could modulate responses during inflammatory and autoimmune processes later in life (349). Indeed, besides the interaction with dietary antigens (as we discussed above), early-life maturation of the immune system and the development of immunological tolerance are achieved through immense pressure from microbial stimulation in our surroundings (349, 350), where microbial influences create a balance between protection and tolerance (351). For instance, commensal microbial colonization limits the expansion of pathogenic/disease-causing pathobionts (352, 353), and more importantly, colonization of commensal microbes early in life can induce Treg cells on mucosal sites (such as the lungs) and subsequently promote homeostasis and tolerance later in life (354, 355). In contrast, microbial depletion or reduced microbiota diversity [e.g., through antibiotic treatment early in life (356)] can have long-term immunological consequences (357, 358), where the lack of microbial diversity during the neonatal stage could lead to a higher incidence of chronic inflammatory and topical disorders later in life (359).

Cases of hyperinflammatory responses that could fit within the criteria for autoimmune disorders have been reported following COVID-19 infections (360–366). This leads us to the question of whether the viral infection itself could contribute to the breakdown of self-tolerance. It is widely accepted that infections could trigger autoimmunity (367–372). Mechanisms of such include (1) bystander activation of innate cells enhancing the presentation of self-antigens and thereby the expansion of autoreactive adaptive counterparts, (2) release of autoantigens, neoantigen formation, and epitope spreading following excessive tissue damage, thus promoting autoimmunity, and (3) induction of molecular mimicry or cross-reactivity when both infectious/exogenous antigens and self-antigens share sequence or structural similarities leading to autoreactivity against self (367, 373). In this regard, although data are still needed on the ability of SARS-CoV-2 to induce bystander activation and neoantigen formation, the non-specific activation of innate immune cells generating a wide array of pro-inflammatory cytokines (e.g., cytokine storms) following COVID-19 infection (374) may lead to tissue damage and generation of neoantigens or epitope spreading, thus initiating autoimmunity. Consistent with this notion, it was previously reported that in severe acute respiratory syndrome-associated coronavirus (SARS-CoV) infection, the presence of cross-reactive epitopes on SARS-CoV spike protein domain 2 could generate antibodies that also cross-reacted with epitopes on lung epithelial cells (375). Similarly, recent reports have shown that based on transcriptomic analysis, SARS-CoV-2 shares molecular similarities with diverse human central nervous system (CNS) protein epitopes that could trigger CNS autoimmunity (376).

On the other hand, infections could be a double-edged sword where different hypotheses exist on how infections could modulate autoimmunity. As postulated by the hygiene hypothesis, infections could counteract autoimmune development and represent a therapeutic intervention against autoimmunity. Notably, there are compelling evidences that specific pathogens suppress different autoimmune conditions in murine models of SLE (377), CNS autoimmunity (378) and T1D (379). Generally, the potential mechanisms that might explain the protective influence of infections on autoimmunity [as reviewed by others (44)] include (1) antigenic competition where a strong immune response is induced by a stronger antigenic stimulus from an infectious agent competes on signals of inflammation (e.g., pro-inflammatory cytokines) with a weaker signal from autoantigens leading to dampened responses towards the weaker stimulus (380), (2) desensitization of antigen recognition receptors such as TLRs due to repeated low-dose antigenic stimulation leading to anergic responses to autoantigens (381–384), and (3) induction of immunosuppressive phenotype or bystander suppression where infectious agents enhance the signaling that rebalances the regulatory to pro-inflammatory T-cell subsets by increasing the Treg cells and limiting Th17 expansion (385).

Furthermore, based on the hygiene hypothesis, or the microbiome depletion theory, there is a positive epidemiological association between the increase of countermeasures to limit infections (e.g., antibiotics, vaccinations, and sanitation strategies) and the incidence of autoimmune and allergic conditions (45). During the COVID-19 pandemic, the worldwide increase of sanitation strategies and the use of numerous types of disinfectants and household products raise concerns around their implications on immune health, especially with the concomitant emergence of allergic and autoimmune conditions (386). Could hygiene measures and specifically the use of disinfectants affect the microbial and immunological modulation of autoimmunity? Although studies investigating the immunopathogenic potential of disinfectants are limited, here we briefly propose three possible mechanisms of how disinfectants could modulate immunity and contribute to autoimmune conditions such as SLE. These include their effects on (1) microbiota diversity, (2) immune cell phenotype and fate, and (3) epigenetic modifications.

Hygiene can disrupt microbiota diversity (387–389), and a positive correlation has been found between microbiota dysbiosis and the increase of autoimmune conditions such as IBD (390–392) and SLE (393–397). This concern is escalating due to the current COVID-19 pandemic that witnessed a dramatic increase in hygiene practices including the use of detergents and disinfectants (398–400). In fact, these control measures may have long-term consequences on the human microbiome (401, 402). Importantly, a recent report suggests that a disturbed gut microbiota might lead to more severe inflammation in COVID-19, where they found positive correlations between microbial dysbiosis and disease severity as well as inflammatory mediators in COVID-19 patients (403).

The use of disinfectants is well established to alter microbiota diversity and microbial load. Chlorine, which is widely used as a water disinfectant, produces byproducts called trihalomethanes (THMs) that are intestinally absorbed and known to perturb the gut microbiota leading to the elevated relative abundance of *Bacteroidetes* and a dose-dependent decrease in the ratio of *Firmicutes* to *Bacteroidetes* (404). Similarly, a reduced *Firmicutes*/*Bacteroidetes* ratio is also evident in different autoimmune diseases (405, 406) including SLE (407). The active chemicals in commercial disinfectants and hand sanitizers (e.g., Triclosan, 5-chloro-2-(2,4-dichloro phenoxy) phenyl) could have a profound impact on the gut microbiome (408, 409), reducing microbiota diversity (410, 411) and increasing the abundance of *Lachnospiraceae* (412). As mentioned earlier, there is a strong correlation between disrupted microbial communities and systemic autoimmune disease pathogenesis. For instance, in MRL/*lpr* mice, reduced *Lactobacillaceae* and increased *Lachnospiracea*e are associated with lupus onset and progression (413). Thus, COVID-19 related hygiene practices, such as the overuse of disinfectants, could indirectly contribute to autoimmunity through inducing microbiota dysbiosis.

Secondly, the use of disinfectants could also tone the function and fate of immune cells and subsequently shape the outcome of autoimmune disease. We have shown that quaternary ammonium compounds (QACs) in widely used antiseptics and surface disinfectants (414) can impair innate immune cell functions (415). QACs increase the production of pro-inflammatory cytokines from murine macrophages *in vitro* and paradoxically impair their phagocytic potential (415). Since prompt macrophage responses are crucial for efferocytosis (phagocytosis of dying cells in an inflammatory milieu) as well as effective clearance of autoantigens, these disinfectants could contribute to autoimmunity *via* impairing macrophages responses (416, 417). Using MRL/*lpr* mice, we found that ambient exposure to QACs hindered the migration of bone marrow-derived neutrophils towards inflammatory stimuli and decreased their infiltration into the lymph nodes. In parallel, QACs upregulated splenic neutrophil expression of checkpoint protein programmed death-ligand 1 (PD-L1). Moreover, QAC exposure dampened the activation of splenic T cells and increased apoptosis of effector T-cell populations, thereby mitigating SLE-associated lymphadenopathy in this mouse model (418). Furthermore, while the phenotype of reduced splenomegaly and lymphadenopathy is an indication of protection against a mouse model of SLE, our findings also indicate that even ambient exposure to QACs could alter neutrophil and T-cell phenotypes, functions, and their fate, raising concerns about the immunotoxicity of these chemicals.

Thirdly, disinfectants may reprogram immune cell functions through epigenetic alteration, thus leading to autoimmunity. For instance, long-term exposure to the chlorine byproducts THMs in drinking water results in global DNA hypomethylation as well as *c-Jun* gene-specific hypomethylation (419). Inhibition of DNA methylation in differentiating Th cells deviates their cytokine responses towards a pro-inflammatory IFNγ^+^ phenotype (420). In addition, a low DNA methylation level in mature T cells could result in T-cell autoreactivity associated with idiopathic SLE (421). These findings on disinfectants and epigenetics warrant further investigations to elaborate their roles in breaking immune homeostasis to promote autoimmunity.

Importantly, while authorities worldwide are more interested in mitigating the spread of COVID-19 infection, the long-term consequences of hygiene strategies on immune modulation need to be addressed. The Centers for Disease Control and Prevention (CDC) recommends to the use of the United States Environmental Protection Agency (EPA) list N-approved disinfectants to combat COVID-19 infection; if the disinfectants on the list are not available, then the bleach solution (Chlorine) is recommended. Notably, QAC-based disinfectants are on the top of the EPA list N. Again, the CDC recommendations raise the concern of whether the overuse of these chemicals by the public could pave the way to autoimmunity as the comet tail of the current pandemic. As we have discussed, QACs and chlorine byproducts have the potential to modulate immune cell fate and induce epigenetic modification, respectively, to break self-tolerance, thus leading to autoimmunity.

## Summary

During the recent decades, improved socio-economic levels have led to modernized dietary and hygiene approaches that are concomitant with an increased prevalence of autoimmune conditions. This suggests the strong influence of environmental factors on immune modulation. Throughout this review, we discussed how dietary components and hygiene could have diverse implications on immune health and importantly, their implication on immune tolerance and autoimmunity. This includes both direct and indirect effects on immune cell programming. Direct effects are those on cell signaling, cell metabolism and energy intake, and epigenetic modification; and indirect effects include those modulating antioxidant capacities and gut microbiota. The emergence of the COVID-19 pandemic has greatly impacted our dietary and hygiene behaviors. It is thus important to consider the immunomodulatory capacities of environmental factors especially for patients suffering from both COVID-19 and autoimmune disease. Future studies will unravel much-needed mechanistic insights on the immune modulation induced by diet and hygiene and lead to more effective management strategies for autoimmune diseases during emerging threats such as COVID-19.

## Author Contributions

Both authors listed have made a substantial, direct, and intellectual contribution to the work.

## Funding

Preparation of this manuscript was supported by the NIH/NIAMS under award number R01-AR073240

## Conflict of Interest

The authors declare that the research was conducted in the absence of any commercial or financial relationships that could be construed as a potential conflict of interest.

## Publisher’s Note

All claims expressed in this article are solely those of the authors and do not necessarily represent those of their affiliated organizations, or those of the publisher, the editors and the reviewers. Any product that may be evaluated in this article, or claim that may be made by its manufacturer, is not guaranteed or endorsed by the publisher.
